# Readability Assessment of Online Patient Education Material on Congestive Heart Failure

**DOI:** 10.1155/2017/9780317

**Published:** 2017-06-01

**Authors:** Akhil Kher, Sandra Johnson, Robert Griffith

**Affiliations:** Geisinger Commonwealth School of Medicine, Scranton, PA, USA

## Abstract

**Background:**

Online health information is being used more ubiquitously by the general population. However, this information typically favors only a small percentage of readers, which can result in suboptimal medical outcomes for patients.

**Objective:**

The readability of online patient education materials regarding the topic of congestive heart failure was assessed through six readability assessment tools.

**Methods:**

The search phrase “congestive heart failure” was employed into the search engine Google. Out of the first 100 websites, only 70 were included attending to compliance with selection and exclusion criteria. These were then assessed through six readability assessment tools.

**Results:**

Only 5 out of 70 websites were within the limits of the recommended sixth-grade readability level. The mean readability scores were as follows: the Flesch-Kincaid Grade Level (9.79), Gunning-Fog Score (11.95), Coleman-Liau Index (15.17), Simple Measure of Gobbledygook (SMOG) index (11.39), and the Flesch Reading Ease (48.87).

**Conclusion:**

Most of the analyzed websites were found to be above the sixth-grade readability level recommendations. Efforts need to be made to better tailor online patient education materials to the general population.

## 1. Introduction

The readily accessible Internet has become the most popular educational resource for the general patient population [1–3]. Patients have turned to consult the search engine Google for diagnoses and treatment of their own health conditions before their primary physician [4]. While having a nearly unlimited knowledge base can be empowering, the unfiltered nature of the Internet can result in patient misinformation and anxiety due to medical jargon and difficult readability of the patient education materials. Guidelines set forth by American Medical Association (AMA) and the US Department of Health and Human Services (USDHHS) dictate that patient reading material should be no higher than a fifth- or sixth-grade reading level in order to be more accessible and comprehensible to the general public [5].

Readability is defined through various formulas based on sentence length, word familiarity, syllables, and other factors via scores that identify a grade level needed to attain to comprehend the presented information. Many recently published articles show that medical websites are not pitched to the appropriate communication levels of the general public [6–10]. In this cross-sectional study, online patient education materials on the particular topic of congestive heart failure (CHF) were assessed by the authors. It is estimated that 5.7 million Americans suffer from CHF, and about half of those afflicted will die within five years of the diagnosis [11]. Early intervention and treatment can improve quality and length of life for most patients. Thus, it is crucial that online information pertinent to CHF be tangible to the general public in order to understand, manage, and track their condition in the appropriate manner. This study focused on assessing the readability levels and reading ease of online CHF articles available to the general public via Google.

## 2. Methods

### 2.1. Search Engine

The Google search engine was used because the majority of patients that use the Internet for health-related information reported using Google [12, 13]. The search term “congestive heart failure” was entered into a Google Chrome web browser. The search was performed on November 29, 2016.

### 2.2. Inclusion and Exclusion Criteria

The first 100 search results were analyzed to determine if they would be eligible for inclusion. Websites were eligible for inclusion if they (1) were in English, (2) were free to access, and (3) provided information on CHF. Websites were excluded if they were advertisements for medical products or news articles or pertained only to animal-based diseases. This caused 30 results to be excluded, leaving 70 websites to be analyzed (see Table 1 for the list of websites included).

### 2.3. Readability Assessment

The readability of each website was assessed using five readability formulas (Table 2).

The Flesch-Kincaid Grade Level (FKGL) and the Flesch Reading Ease (FRE) are both calculated using the average sentence length (i.e., the number of words divided by the number of sentences) and the average syllables per word (i.e., the number of syllables divided by the number of words) using different formulas [14, 15].

The Gunning-Fog Score (GFS) is calculated using the average sentence length and the number of polysyllabic words (i.e., those with three or more syllables) [16]. The counted polysyllabic words do not include (i) proper nouns, (ii) combinations of hyphenated words, or (iii) two-syllable verbs made into three with -es and -ed endings.

The Coleman-Liau Index (CLI) is calculated using the average number of letters per 100 words and the average sentence length [17]. Unlike the other four readability tests, the CLI does not assess the number of syllables in a given text.

The Simple Measure of Gobbledygook (SMOG) index is calculated using the number of polysyllabic words in three ten-sentence samples near the beginning, middle, and end of a piece of text [18]. If there are fewer than 30 sentences, the formula contains a factor to correct for this.

The five readability tests chosen have been widely used in a variety of previous studies. Each test assesses readability according to word difficulty and sentence length using different weighting factors. Five different readability tests were used in order to compare the readability of each website based upon different factors. The FRE is a 100-point scale with higher scores indicating more easily understood text (Table 3). The remaining four measures, FKGL, GFS, CLI, and SMOG, indicate the US academic grade level (number of years of education) necessary to comprehend the written material. For example, a score of 13.5 would indicate a grade level appropriate for a first year undergraduate student, while 6 would indicate that the available health information can be comprehended by an individual who is in or has completed the sixth grade. To prevent human error during calculations and for ease of use, a single online readability calculator recommended by the National Institutes of Health (NIH) was used for all five readability tests [19, 20].

Prior to analyzing the data, the “ideal” criteria for the readability of the online resources were established. The USDHHS recommends health materials to be written at the 5th- or 6th-grade level to ensure wide understanding. Thus, the level of acceptable readability was determined to be greater than or equal to 80.0 for the FRE and less than or equal to 6.9 for the FKGL, GFS, CLI, and SMOG.

### 2.4. Statistical Analysis

Standard data entry and analysis were done using a Microsoft Excel spreadsheet. Independent upper-tailed hypothesis tests were conducted for each readability index. Results were considered statistically significant at a *p* value of 0.05 or less.

## 3. Results

Of the 100 websites identified, only 70 met the study inclusion criteria and were analyzed for readability. Thirty websites were excluded because they did not describe CHF (14), pertained to animal-based diseases (7), were advertisements (4), required payment (3), or were news articles (2).

**Figure 1:**
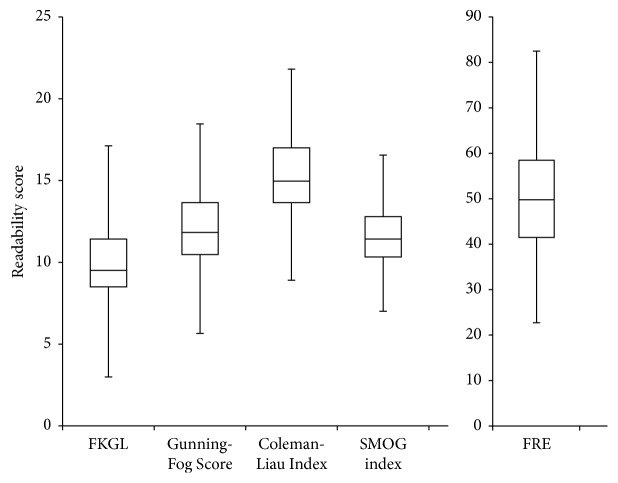
Box-and-whisker plots showing median readability scores of health information websites (*n* = 70; *p* < 0.05).

All five assessment tools reported statistically significant results in which *p* value was less than the standard alpha value of 0.05. The distribution of each readability score for all of the websites evaluated is summarized in Table 4. A comparison of mean readability scores between general medical websites and specialty-specific websites is shown in Table 5. Of the 70 websites, only 5 (7.1%) of them were within the limits of the recommended sixth-grade reading level on at least one assessment tool (Table 6). No websites were at or under the sixth-grade reading level using all five assessment tools.  Figure 1 details the median scores of the health information websites using a box-and-whisker plot.

## 4. Discussion

Internet access has opened up a plethora of resources to use as education materials but the writing style and jargon of most medically relevant articles favor a small percentage of the general public. In order to prevent confusion, undue stress, and misinformation, it is important for patients to have adequate and appropriate medical information available to them in all healthcare settings. However, the material presented online cannot be utilized effectively if it is presented in a style that is beyond the scope of the general population. One study showed that about 1 in 5 patients has utilized the Internet for obtaining medical information, however, the majority of them encountered difficulties comprehending the information available to them [21]. The guidelines set forth by the AMA and USDHHS state that the information must be written at or below a sixth-grade reading level in order to be accessible to the public. The aim of this study is elucidate the readability of available online health-related information in terms of these standard guidelines. This study focused specifically on congestive heart failure.

### 4.1. Online Health Information Readability

The search engine Google was used to assess the readability of websites relevant to congestive heart failure. Of the first 100 search results, 70 fit the inclusion criteria of this study and were consequently analyzed via five readability assessment tools. As the results indicate, 92.9% of the CHF websites assessed were above the recommended levels. As Table 5 indicates, there was no significant difference in the readability scores of general medical websites as compared to specialty-specific websites. Only five websites fell within the recommended levels but none of them passed all five assessment tools, further portraying that medical articles found online are not written at an appropriate level for the average US citizen. This was seen across both general medical websites and diagnosis-specific websites.

In the case of congestive heart failure, it is crucial for patients to understand, manage, and track their health condition to improve their quality and longevity of life. To our knowledge, this is one of few studies to use these five readability assessment tools to assess the readability of online patient education information relating specifically to congestive heart failure. The readability of web-based literature has been assessed in many healthcare arenas such as colorectal surgery, ophthalmology, dermatology, nephrology, orthopedics, psychiatry, and endocrinology [5, 22–27].

Hutchinson et al. used four of the five readability indices used in our study on websites that were also included and assessed in our study [28]. According to the prior study, the average readability of Wikipedia.org, MayoClinic.org, WebMD.com, Medicine.net, and NIH.gov on the disease-specific topic of congestive heart failure was found to be above the recommended sixth-grade reading level. This was consistent with the findings in our study when the average of all five of the readability assessment tools is taken. In both studies, the NIH website had the lowest average reading grade level of the five websites. Tulbert et al. also analyzed Wikipedia.org and WebMD.com using the FKGL and FRE readability tests and likewise found that both websites were above the sixth-grade reading level [23].

Another study analyzed a broad spectrum of websites relating to 16 medical specialties using ten readability indices, including the five used in this study [10]. According to that study, the American Academy of Family Physicians (AAFP) website was written above the recommended sixth-grade readability level using the FKGL, FRE, GFS, CLI, and SMOG. Our study also utilized the AAFP website for the more disease-specific topic of congestive heart failure and found that it was written above the recommended reading level in all five tests.

### 4.2. Health Literacy

Patient education is an integral part of the physician-patient relationship. However, a majority of US citizens are known to have limited health literacy [29, 30]. There have been several efforts undertaken to provide a profile of health literacy skills of specific patient populations that have found striking evidence of inadequate literacy skills, including in medical care settings [31].

Low health literacy has been found to negatively impact health and well-being. Low health literacy rates have been correlated with higher mortality in the elderly and impose a higher risk of living with chronic illnesses [29]. On an individual level, this results in preventable recurrent hospitalizations or clinic visits. On the national level, it has been found that inadequate health literacy costs the US economy between $106 and $236 billion dollars annually. Health literacy has long been recognized as one of the central challenges we all face in American healthcare [32]. We can take the necessary steps in mitigating this issue by following recommendations to improve the readability of online health information.

### 4.3. Recommendations

It is important to adhere to the AMA and USDHHS guidelines in keeping patient education websites at a sixth-grade readability level or below in order to broaden the patient base that the information can reach. By doing so, more patients will be able to find the appropriate information on websites, be able to understand what they found, and be able to act appropriately on that understanding [33].

According to the NIH, this is particularly important for the first few lines of text because if the reader encounters difficulty with a passage at the beginning, they may stop reading altogether [19]. The Institute for Healthcare Improvement recommends using simpler words, shorter sentences, and avoiding medical jargon, all of which will also serve to improve website readability scores [34]. Websites with scores that do not adhere to the AMA and USDHHS guidelines should consider rewriting materials to aim for a grade level less than or equal to 6.9 and FRE scores below 60. These changes should ensure a simple, quick, and cost-effective effort to make a definitive change that will improve the readability of available online health-related information for the general public.

### 4.4. Limitations

This study has a few important limitations. Only the first 100 results were reviewed in this study and only with one search phrase. This is only a portion of all the available websites on our topic of interest, although we found that using related search terms resulted in very similar results. In this study, we used only one search engine for retrieving information; however, the search engine we used is the most widely used engine globally for obtaining health-related information [7, 35].

The scope of this study is focused particularly on US English-speaking patient populations. Websites were excluded if they were non-English, and thus the results may not be applicable to a non-English-speaking patient population. The results obtained may be location-specific because search results will vary based on the server used; thus it is difficult to draw more general conclusions about the entire global patient population. Additionally, the readability indices that were utilized were originally created to gauge the readability of English texts using US grade levels. However, the authors of this study acknowledge that the data results may be extrapolated and applied to English-speaking patient populations outside the US, in which the readability grade level can be considered the number of years of formal education conducted in English.

As a cross-sectional study, we acknowledge that our search results reflect a snapshot in time from a single location and does not represent or account for every patient's search experience. The available resources on the Internet are always growing and changing, and thus search results retrieved at different moments in time may differ. The results of our study are meant to instigate reflection, to initiate efforts by website authors to improve readability, and to serve as a comparison point for reassessment in the future.

## 5. Conclusion

This study showed that the current readability of websites pertaining to congestive heart failure was poor. The Internet has become a powerful, accessible resource for many patients to use for their own medical management and comprehension. However, many patient education websites pose material at a reading level that is not suitable for the average adult, causing readability as well as comprehension to suffer. Poor health literacy has been found to negatively impact health and even inflate healthcare costs in the United States. It is therefore imperative to scrutinize the Internet resources available to the general populace in order to prevent mismanagement and subpar healthcare outcomes. We have highlighted easy, cost-effective methods such as using shorter sentences and limiting medical jargon in order to better achieve the recommended readability level. It is our recommendation that patient education websites be reevaluated for adherence to readability guidelines set forth by the NIH and USDHHS in order to ensure that resources are inclusive to a wider audience. Through this study, we hope to make website creators aware of the utility of readability indices to achieve this goal.

## Figures and Tables

**Table 1:** List of congestive heart failure websites with their Google ranking and website type.

Rank	Website Uniform Resource Locator (URL)	Website Type
1	http://www.webmd.com/heart-disease/guide-heart-failure#1	General
2	http://www.medicinenet.com/congestive_heart_failure_chf_overview/article.htm	General
3	https://en.wikipedia.org/wiki/Heart_failure	General
4	http://www.mayoclinic.org/diseases-conditions/heart-failure/basics/definition/con-20029801	General
5	http://www.healthline.com/health/congestive-heart-failure	General
6	http://www.heart.org/HEARTORG/Conditions/CongenitalHeartDefects/TheImpactofCongenitalHeartDefects/Congestive-Heart-Failure-and-Congenital-Defects_UCM_307111_Article.jsp#.WD4K8LIrLIU	Specialized
7	https://medlineplus.gov/heartfailure.html	General
8	http://emedicine.medscape.com/article/163062-overview	General
9	https://www.ncbi.nlm.nih.gov/pubmedhealth/PMHT0022300/	General
10	https://www.merckmanuals.com/professional/cardiovascular-disorders/heart-failure/heart-failure	General
11	http://www.emedicinehealth.com/congestive_heart_failure/article_em.htm	General
12	https://www.cardiosmart.org/Heart-Conditions/Heart-Failure	Specialized
13	https://www.cincinnatichildrens.org/health/c/chf	General
14	https://www.acls.net/guide-to-congestive-heart-failure.htm	Specialized
15	http://www.hopkinsmedicine.org/heart_vascular_institute/conditions_treatments/conditions/congestive_heart_failure.html	General
16	http://www.heartsite.com/html/chf.html	Specialized
17	https://labtestsonline.org/understanding/conditions/chf/	General
18	http://www.aafp.org/afp/topicModules/viewTopicModule.htm?topicModuleId=26	General
19	https://www.ghc.org/healthAndWellness/?item=/common/healthAndWellness/conditions/heartDisease/chfBasics.html	General
20	http://www.heartpoint.com/congheartfailure.html	Specialized
21	https://getpalliativecare.org/whatis/disease-types/congestive-heart-failure-palliative-care/	General
22	http://www.rxlist.com/script/main/art.asp?articlekey=6972	General
23	http://www.lifelinescreening.com/What-We-Do/What-We-Screen-For/Congestive-Heart-Failure	General
24	https://www.franciscanhealth.org/diseases-and-conditions/congestive-heart-failure	General
25	https://www.ahn.org/specialties/cardiovascular-institute/heart-failure	General
26	http://www.healio.com/cardiology/learn-the-heart/cardiology-review/systolic-congestive-heart-failure	Specialized
27	http://www.texasheart.org/HIC/Topics/Cond/CHF.cfm	Specialized
28	https://www.caring.com/articles/congestive-heart-failure	General
29	http://www.healthcommunities.com/congestive-heart-failure/chf-overview.shtml	General
30	http://www.healthcentral.com/encyclopedia/hc/congestive-heart-failure-3169011/	General
31	https://www.uabmedicine.org/patient-care/conditions/heart-failure	General
32	http://www.vistahealth.com/vista-health-system/services/vista-health-system-congestive-heart-failure-2480.aspx	General
33	http://www.lifeextension.com/protocols/heart-circulatory/congestive-heart-failure/page-01	General
34	http://www.ithacajournal.com/story/sponsor-story/cayugamed/2016/11/27/cayuga-med-congestive-heart-failure/93292768/	General
35	http://www.utswmedicine.org/conditions-specialties/heart/programs/congestive-heart-failure/	General
36	http://www.dovemed.com/diseases-conditions/congestive-heart-failure/	General
37	https://www.childrensmn.org/references/pfs/condill/congestive-heart-failure.pdf	General
38	https://www.dukehealth.org/treatments/heart/congestive-heart-failure	General
39	http://heartfailurecenter.com/hfcheartfailurestages.shtm	Specialized
40	http://www.wakemed.org/heart-vascular-congestive-heart-failure	General
41	https://demanddeborah.org/medical-services/congestive-heart-failure/	General
42	https://www.vanderbilthealth.com/heart/14221	General
43	http://www.hfsa.org/what-is-heart-failure/	Specialized
44	http://www.everydayhealth.com/congestive-heart-failure/guide/	General
45	https://www.ucsfhealth.org/conditions/heart_failure/signs_and_symptoms.html	General
46	https://www.healthgrades.com/conditions/congestive-heart-failure--symptoms	General
47	https://www.cdc.gov/dhdsp/data_statistics/fact_sheets/fs_heart_failure.htm	General
48	http://www.umm.edu/health/medical/reports/articles/heart-failure	General
49	https://www2.nau.edu/~daa/lecture/chfmeds.htm	General
50	http://www.baylorhealth.com/SpecialtiesServices/HeartHealth/patientandfamilysupport/Pages/CongestiveHeartFailureClinic.aspx	General
51	https://www.rush.edu/services/conditions/congestive-heart-failure	General
52	http://www.fpnotebook.com/cv/chf/CngstvHrtFlr.htm	General
53	https://www.drweil.com/health-wellness/body-mind-spirit/heart/congestive-heart-failure-chf/	General
54	http://www.mylvad.com/congestiveheartfailure/advanced-congestive-heart-failure	Specialized
55	http://www.childrenshospital.org/conditions-and-treatments/conditions/congestive-heart-failure	General
56	https://tricare.mil/CoveredServices/BenefitUpdates/Archives/10_19_16_CHF	General
57	http://www.umcvc.org/conditions-treatments/heart-failure	Specialized
58	https://link.springer.com/referenceworkentry/10.1007%2F978-0-387-79948-3_2171	General
59	http://health.howstuffworks.com/diseases-conditions/cardiovascular/heart/congestive-heart.htm	General
60	https://www.bayhealth.org/congestive-heart-failure	General
61	http://www.mauryregional.com/our-services/heart-and-stroke-services/heart/congestive-heart-failure	General
62	http://www.rcjournal.com/contents/04.06/04.06.0403.pdf	General
63	http://www.inovaheart.org/heart-care/heart-failure	Specialized
64	http://www.kh.org/site/c.dkLSK7OPLnKaE/b.8326805/k.56CA/Congestive_Heart_Failure.htm	General
65	https://patient.info/health/heart-failure-leaflet	General
66	https://www.lourdes.com/centers-and-services/congestive-heart-failure-chf/	General
67	http://www.hospitalmedicine.org/Web/Quality___Innovation/Implementation_Toolkit/CHF/CHF_overview.aspx	General
68	http://www.chw.org/medical-care/herma-heart-center/conditions/congestive-heart-failure/	General
69	http://connectedhealth.partners.org/patient-programs/remote-monitoring/heart-failure.aspx	General
70	https://www.growthhouse.org/chfcopd.html	General

**Table 2:** Readability test formulas used to analyze patient education websites.

Readability test	Formula
Flesch-Kincaid Grade Level	$FKGL=0.39\left(\frac{total\;\;words}{total\;\;sentences}\right)+11.8\left(\frac{total\;\;syllables}{total\;\;words}\right)-15.59$
Flesch Reading Ease	$FRE=206.835-1.015\left(\frac{total\;\;words}{total\;\;sentences}\right)-84.6\left(\frac{total\;\;syllables}{total\;\;words}\right)$
Gunning-Fog Score	$GFI=0.4\left[\left(\frac{total\;\;words}{total\;\;sentences}\right)+100\left(\frac{complex\;\;words}{total\;\;words}\right)\right]$
Coleman-Liau Index	CLI = 0.588*L* − 0.296*S* − 15.8
SMOG Index	$SMOG=1.043\sqrt{number\;\;of\;\;complex\;\;words\times \frac{30}{total\;\;sentences}+3.1291}$

*Variables*. Average number of letters per 100 words (*L*), average number of sentences per 100 words (*S*).

FKGL, Flesch-Kincaid Grade Level; FRE, Flesch Reading Ease; GFS, Gunning-Fog Score; CLI, Coleman-Liau Index; SMOG, Simple Measure of Gobbledygook.

**Table 3:** Flesch reading ease scores with equivalent US education level and USDHHS readability rating.

FRE score	Equivalent education level	USDHHS readability
0–29	College graduate	Difficult
30–49	College	
50–59	10th–12th	

60–69	8th-9th	Average
70–79	7th	

80–89	6th	Easy
90–100	5th	

FRE, Flesch Reading Ease; USDHHS, US Department of Health and Human Services.

**Table 4:** Mean readability scores of health information websites.

Readability test	Mean score	Standard deviation	*p* value
FKGL	9.79	2.28	0.0483
GFS	11.95	2.84	0.018
CLI	15.17	2.67	0.0003
SMOG	11.39	2.1	0.005
FRE	48.87	13.29	0.0096

**Table 5:** Mean readability scores of general medical websites as compared with specialty websites.

Readability test	General	Specialty	*p* value
FKGL	9.93	9.08	0.339
GFS	12.03	11.61	0.4329
CLI	15.62	13.16	0.1893
SMOG	11.41	11.23	0.4609
FRE	47.12	56.77	0.2647

**Table 6:** Category breakdown of readability scores of health information websites.

Readability scores	Number of websites (*n* = 70)
FRES	
Easy (80–100)	1
Average (60–79)	13
Difficult (0–59)	56
FKGL	
Up to grade 6	3
Grades 6–10	48
Beyond grade 10	19
GFS	
Up to grade 6	1
Grades 6–10	20
Beyond grade 10	49
CLI	
Up to grade 6	0
Grades 6–10	4
Beyond grade 10	66
SMOG	
Up to grade 6	0
Grades 6–10	13
Beyond grade 10	57
